# Identification of 11-Hydroxytephrosin and Torosaflavone A as Potential Inhibitors of 3-Phosphoinositide-Dependent Protein Kinase 1 (PDPK1): Toward Anticancer Drug Discovery

**DOI:** 10.3390/biology11081230

**Published:** 2022-08-18

**Authors:** Akhtar Atiya, Fahad A. Alhumaydhi, Sharaf E. Sharaf, Waleed Al Abdulmonem, Abdelbaset Mohamed Elasbali, Maher M. Al Enazi, Anas Shamsi, Talha Jawaid, Badrah S. Alghamdi, Anwar M. Hashem, Ghulam Md. Ashraf, Moyad Shahwan

**Affiliations:** 1 Department of Pharmacognosy, College of Pharmacy, King Khalid University (KKU), Guraiger St., Abha 62529, Saudi Arabia; 2 Department of Medical Laboratories, College of Applied Medical Sciences, Qassim University, Buraydah 52571, Saudi Arabia; 3 Pharmaceutical Chemistry Department, College of Pharmacy Umm Al-Qura University, Makkah 21961, Saudi Arabia; 4 Clinical Research Administration, Executive Administration of Research and Innovation, King Abdullah Medical City in the Holy Capital, Makkah 21955, Saudi Arabia; 5 Department of Pathology, College of Medicine, Qassim University, P.O. Box 6655, Buraidah 51452, Saudi Arabia; 6 Department of Clinical Laboratory Science, College of Applied Sciences-Qurayyat, Jouf University, Sakaka 72388, Saudi Arabia; 7 Department of Medical Laboratory Sciences, College of Applied Medical Sciences, Prince Sattam Bin Abdelaziz University, Al-Kharj 11942, Saudi Arabia; 8 Center of Medical and Bio-Allied Health Sciences Research, Ajman University, Ajman P.O. Box 346, United Arab Emirates; 9 Center for Interdisciplinary Research in Basic Sciences, Jamia Millia Islamia, Jamia Nagar, New Delhi 110025, India; 10 Department of Pharmacology, College of Medicine, Al Imam Mohammad Ibn Saud Islamic University (IMSIU), Riyadh 13317, Saudi Arabia; 11 Neuroscience Unit, Department of Physiology, Faculty of Medicine, King Abdulaziz University, Jeddah 22254, Saudi Arabia; 12 Pre-Clinical Research Unit, King Fahd Medical Research Center, King Abdulaziz University, Jeddah 22254, Saudi Arabia; 13 Department of Medical Microbiology and Parasitology, Faculty of Medicine, King Abdulaziz University, Jeddah 22254, Saudi Arabia; 14 Vaccines and Immunotherapy Unit, King Fahd Medical Research Center, King Abdulaziz University, Jeddah 22254, Saudi Arabia; 15 Department of Medical Laboratory Sciences, Faculty of Applied Medical Sciences, King Abdulaziz University, Jeddah 22254, Saudi Arabia; 16 College of Pharmacy, Ajman University, Ajman P.O. Box 346, United Arab Emirates

**Keywords:** 3-phosphoinositide-dependent protein kinase 1, cancer, Indian phytoconstituents, drug discovery, virtual screening, conformational dynamics

## Abstract

**Simple Summary:**

Cancer is amongst the leading cause of mortality across the globe. Thus, researchers are continuously working in the field of cancer therapeutics. Understanding the complexities of the metabolic switching of cancer cells will aid in developing novel ways for successful and targeted therapy. Some proteins are overexpressed in different types of cancers and thus, our research is aimed at identifying inhibitors of these proteins. Natural compounds due to their minimal side effects coupled with broad therapeutic potential are in focus for these studies. Our method will be valuable in developing cancer therapies that leverage natural leads in this domain.

**Abstract:**

The 3-phosphoinositide-dependent protein kinase 1 (PDPK1) has a significant role in cancer progression and metastasis as well as other inflammatory disorders, and has been proposed as a promising therapeutic target for several malignancies. In this work, we conducted a systematic virtual screening of natural compounds from the IMPPAT database to identify possible PDPK1 inhibitors. Primarily, the Lipinski rules, ADMET, and PAINS filter were applied and then the binding affinities, docking scores, and selectivity were carried out to find effective hits against PDPK1. Finally, we identified two natural compounds, 11-Hydroxytephrosin and Torosaflavone A, bearing substantial affinity with PDPK1. Both compounds showed drug-likeness as predicted by the ADMET analysis and their physicochemical parameters. These compounds preferentially bind to the ATP-binding pocket of PDPK1 and interact with functionally significant residues. The conformational dynamics and complex stability of PDPK1 with the selected compounds were then studied using interaction analysis and molecular dynamics (MD) simulations for 100 ns. The simulation results revealed that PDPK1 forms stable docked complexes with the elucidated compounds. The findings show that the newly discovered 11-Hydroxytephrosin and Torosaflavone A bind to PDPK1 in an ATP-competitive manner, suggesting that they could one day be used as therapeutic scaffolds against PDPK1-associated diseases including cancer.

## 1. Introduction

The 3-phosphoinositide-dependent protein kinase 1 (PDPK1) is the “master kinase of the AGC protein kinase family”, which activates many protein kinase groups including AKT, PKC, and S6K, and encodes a protein of 63,152 Da and plays a crucial role in cancer progression [1,2,3,4]. Activating the phosphorylation to PKB/AKT1 plays a crucial role in transmitting insulin signals to downstream targets that control cell proliferation and survival [5]. It is expressed in most vertebrate tissues, with the highest levels in the cerebellum, testis, spleen, and bone marrow [6]. It also regulates serum/glucocorticoid-regulated kinase 3 for the survival of prostate cancer [4].

PDPK1 is a 556 amino acid residue long polypeptide with two domains (i.e., the kinase and the Preckstrin homology (PH) domain) [7]. The kinase domain lies at the 82–342 aa region, while the PH domain lies at the 459–550 amino acid residues [8]. The PH domain helps PDPK1 bind with phosphatidylinositol (3,4)-bisphosphate and phosphatidylinositol (3,4,5)-trisphosphate and plays a vital role in the localization and import of PDPK1 [7]. Another essential activity of the PH domain is PDPK1 homodimerization [9]. The ATP binding sites of PDPK1 are located at K111, E116, E209 and D223, while D205 serves as the active site (UniProt, the Universal Protein resource, ID: O15530). The nucleotide-binding region on PDK1 lies between the 92–94 and 160–162 amino acid residues. Overall, it is essential for various cellular processes including the activation of the PI3K signaling pathway [10]. The above observations suggest that PDPK1 plays a vital role in oncogenesis. Therefore, PDPK1 is reflected as an attractive molecular target for the discovery of small molecule inhibitors with anticancer potential.

LY333531 (also known as ruboxistaurin) is a macrolactam organic compound and a known pharmacologically proven PDPK1 inhibitor co-crystallized in PDB ID 1UU3 [11]. LY33331 is a member of the bisindolylmaleimide family and is an investigational small therapeutic molecule for diabetic retinopathy and cardiac ventricular hypertrophy complications [12]. LY33331 is a protein kinase C-beta (PKCβ) specific inhibitor and shows an appreciable inhibitory potential toward PDPK1 [11]. Specificity in PDPK1-associated complexities has become a major concern in therapeutics, demanding further research and the development of novel and specific small molecule inhibitors against PDPK1. This study utilized LY333531, a positive control, to identify potential PDPK1 inhibitors.

Virtual screening is one of the most successful techniques for identifying small molecule inhibitors in drug discovery [13,14]. It estimates the likelihood that the ligand will bind to protein with high affinity [15]. In this work, we used 9000 natural compounds from the IMPPAT database, the most extensive database on Indian phytochemicals for virtual screening. We obtained the 3D-structure of PDPK1 from the PDB database (PDB ID: 1UU3) [11]. Next, we screened 9000 compounds by applying the Lipinski rule of five to them and obtained ~6000 compounds. Subsequently, we performed a multi-step screening of 6000 compounds against PDPK1, followed by several other parameters and all-atom molecular dynamics (MD) simulations.

## 2. Methods and Materials

### 2.1. Computer Environment and Web Resources

Bioinformatics tools such as MGL AutoDock tools [16], PyMOL [17], InstaDock [18], and Discovery Studio visualizer [19] were employed for molecular docking-based virtual screening and visualization purposes. Various online servers and resources such as UniProt [20], IMPPAT database [21], SwissADME [22], Pass Online [23], etc. were used in this study for the retrieval and analysis of data.

### 2.2. Receptor Preparation

The structure of human PDPK1 (PDB ID: 1UU3, Resolution: 1.70 Å) was retrieved from the RCSB website (https://www.rcsb.org/structure/1uu3, accessed on 1 March 2022) in the PDB format [11]. The amino acid sequence of PDPK1 was taken from UniProt (https://www.uniprot.org/uniprotkb/O15530/entry, accessed on 1 March 2022) and was used for the remodeling as the parent structure had some missing residues. Self-template-based homology modeling was performed using the MODELLER (version 10.2) software in PyMod 3, a plugin in PyMOL, to fill the missing residues by taking the original structure as a template [24]. The newly generated PDPK1 model was used for further analysis after the required processing for optimization.

### 2.3. Databases Used for Screening

A primary consideration when searching for a compound library for a successful virtual screening program is to have a database with the greatest structural variety possible. This increases the chances of discovering a potential hit with efficacy. Several small-molecule libraries such as the IMPPAT [21], Drug Bank [25], National Cancer Institute (NCI) database [26], PubChem [27], the Binding Database [28], and ZINC library [29] offer a diverse range of chemical compounds. In the present study, we derived a library of Indian medicinal plants from the IMPPAT database containing 9000 phytochemicals [21]. IMPPAT is a free database on Indian plant-based compounds to date, which can be used for virtual screening. The database provides three-dimensional processed chemical structures in the database.

### 2.4. Molecular Docking-Based Virtual Screening

To identify PDPK1-binding molecules with a high affinity, molecular docking-based filtering was used. Docking and interaction analyses were carried out using a variety of bioinformatics applications including InstaDock, PyMOL, and DS Visualizer. The processed PDPK1 structure was utilized as a receptor file, and the IMPPAT database of phytoconstituents was utilized as a ligand source. InstaDock was used to dock using a blind search space where the X, Y, and Z sizes were set to 50 Å, 60 Å, and 60 Å centralized at 81.93 Å, 20.57 Å, and 2.31 Å, respectively. Based on the affinity score, the top docking hits were retrieved, and probable docked conformers were created for interaction investigation. DS Visualizer was used to study the potential interactions between the compounds and the PDPK1 binding pocket. The small molecules interacting with the PDPK1 binding pocket’s critical residues were chosen.

### 2.5. ADMET Properties of Compounds

The pharmacological and pharmacokinetic features of the compounds were calculated using the SwissADME [22] and pkCSM [30] webservers. It compares the input structure with the training set and calculates the probable properties based on different parameters.

### 2.6. PASS Analysis

The PASS website was used to predict the pharmacological and biological properties of the substances [23]. The PASS server analyses a compound’s biological potential based on its structure–activity relationship. The server compares the intended structure to a built-in training set of various biological functions. PASS predicts the probable biological activities based on the ‘probability of being active (Pa)’ to ‘probability of being passive (Pi)’. A greater Pa value indicates that a biological trait for a chemical under investigation is more likely.

### 2.7. MD Simulations

The GROMACS (GROningen MAchine for Chemical Simulations) package was used to run all-atom MD simulations on a HP Z840 computer [31]. To perform simulation on the PDPK1 in free and ligand-bound forms with 11-Hydroxytephrosin and Torosaflavone A, the Gromos force-field was used in the simulation procedure [32]. The topology parameters for 11-Hydroxytephrosin and Torosaflavone A were generated using the PRODRG website (http://davapc1.bioch.dundee.ac.uk/cgi-bin/prodrg, accessed on 22 March 2022). Each complex was housed in a box of cubic boundaries with a 10 Å radius around the perimeter. The SPC16 model was used to supply water molecules for solvation in watery environments [33]. A sufficient amount of counter ions were introduced to ensure that the systems were charged neutral. The steepest descent algorithm was used for all complexes to reduce the energy consumption. After the energy minimization phase, the position restraint operation was performed with the NVT and NPT equilibrium. Finally, a 100 ns simulation run was carried out for each system, and the resulting trajectory was examined using the built-in GROMACS facilities.

### 2.8. Principal Component Analysis and Free Energy Landscapes

Principal component analysis (PCA) has proven to be useful for exposing basic motions in proteins and investigating their folding kinetics [34]. PCA and free energy landscape (FEL) analyses were performed using the essential dynamics technique on the generated simulation trajectories [35]. PCA is a mathematical method that uses a covariance matrix to compress a multidimensional set of variables to a smaller dimension [36]. It helps us examine the conformational sampling of PDPK1 and its complexes with 11-Hydroxytephrosin and Torosaflavone A by diagonalizing the eigenvectors for the covariance matrix. The FEL analysis was carried out to inspect the folding behavior of PDPK1 in free and ligand-bound forms with 11-Hydroxytephrosin and Torosaflavone A.

## 3. Results and Discussion

### 3.1. Molecular Docking-Based Virtual Screening

A total of 6093 compounds were extracted from the IMPPAT database based on the Lipinski’s rule of five. The virtual screening of compounds was undertaken to find high-affinity binding partners of PDPK1. InstaDock was employed for the molecular docking-based virtual screening of all compounds in the filtered library. Log files and out-files containing affinity scores and docked poses were created for each compound in the database. The binding affinity was used as a significant filter to improve the efficiency of discovering effective inhibitors of PDPK1 with novel scaffolds. Based on projected docking scores with PDPK1, the top 50 compounds were selected. The results demonstrate that the selected molecules exhibited significant affinities with PDPK1, ranging from −8.6 to −11.6 kcal/mol (Table 1). The comparative results showed that the selected compounds had higher affinities than LY333531 toward PDPK1. During this screening, we discovered that several of these compounds had a high binding affinity score for the PDPK1 binding pocket, which can be used to further narrow down the search for possible PDPK1 inhibitors.

### 3.2. ADMET Properties of Compounds

Physiological and pharmacokinetic characteristics are critical for the selection and development of drug-like molecules. Compounds that pass the screening for physicochemical and ADMET properties have a screening higher chance of clinical success. The pkCSM website calculated the physicochemical and ADMET parameters of all the compounds selected from the docking filter. For all 50 compounds, different ADMET parameters were determined along with the PAINS filter. This filter chose three compounds with an excellent physicochemical attribute set with a zero PAINS pattern. The computed ADMET characteristics of the selected molecules are shown in Table 2. The selected compounds had better ADMET properties than LY333531, as it showed AMES toxicity in the pkCSM prediction.

### 3.3. PASS Analysis

The PASS server has a comprehensive training set comprising a variety of bioactive chemicals and their structure–activity connections from various clinical and preclinical studies [23]. The PASS server uses the involved training set to predict the biological activity for a chemical compound. The biological activity of the filtered compounds was explored where two compounds, 11-Hydroxytephrosin and Torosaflavone A, were found to pass the PASS biological activity screening. According to the findings, both compounds have anticancer and kinase inhibitory capabilities. When Pa > 0.7, the likelihood of obtaining the expected biological property for a molecule is high. 11-Hydroxytephrosin and Torosaflavone A have been demonstrated when Pa > Pi, high estimates for anticancer, TP53 expression enhancer, and antineoplastic with Pa values ranging from 0.540 to 0.934 (Table 3). LY333531 was also shown to have similar properties to the elucidated compounds, supporting the PASS study.

### 3.4. Interaction Analysis

**Figure 1:**
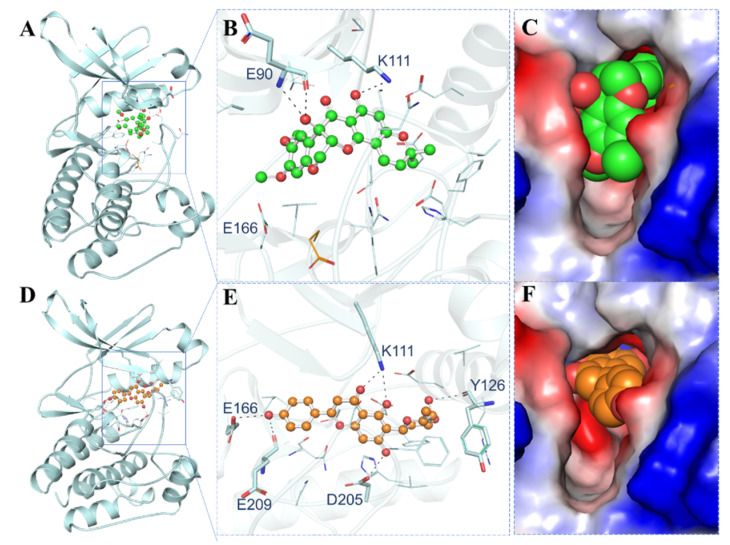
Representation of PDPK1 in complex with the elucidated compounds. (**A**) Cartoon representation of PDPK1 with 11-Hydroxytephrosin. (**B**) Magnified cartoon representation of PDPK1 with 11-Hydroxytephrosin. (**C**) Charged view of PDPK1 binding pocket space-filled by 11-Hydroxytephrosin. (**D**) Cartoon representation of PDPK1 with Torosaflavone A. (**E**) Magnified cartoon representation of PDPK1 with Torosaflavone A. (**F**) Charged view of PDPK1 binding pocket space-filled by Torosaflavone A.

The interaction of the elucidated compounds with PDPK1 was investigated for their probable binding. The docked conformers of 11-Hydroxytephrosin and Torosaflavone A interacted with several common residues during the investigation. The PDPK1 ATP-binding sites Lys111, Glu166, and Glu209 and active site residue Asp205 were found to make direct hydrogen bonds with the elucidated compounds. Figure 1 shows the binding prototypes of 11-Hydroxytephrosin and Torosaflavone A where they have shared common interactions with the crucial residues of the PDPK1 binding pocket (Figure 1B,E). 11-Hydroxytephrosin is bound inside the PDPK1 active site in close proximity and shows complementarity (Figure 1C). Torosaflavone A is docked into the deep cavity of the protein’s binding site to block the ATP-binding pocket of PDPK1 (Figure 1F).

**Figure 2:**
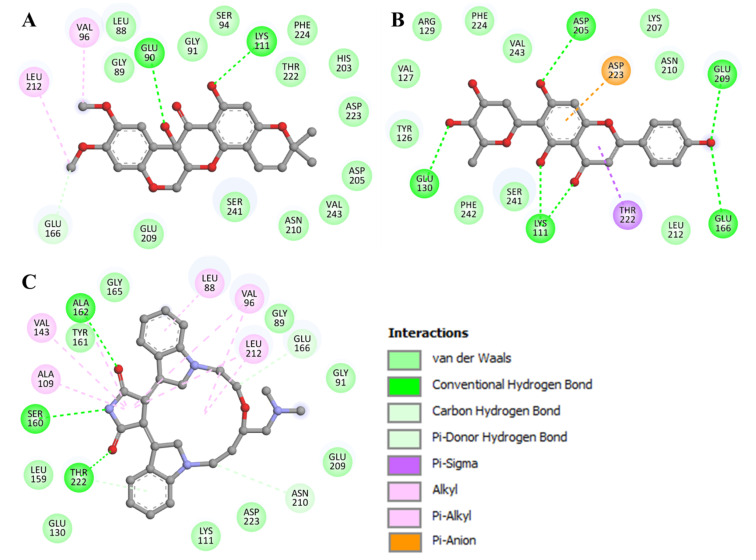
A 2D presentation of PDPK1 residues interacting with (**A**) 11-Hydroxytephrosin, (**B**) Torosaflavone A, and (**C**) LY333531.

Detailed interaction analyses aid in determining the kind and nature of non-covalent interactions. The binding modes and interaction patterns of the selected two compounds were further analyzed utilizing Discovery Studio Visualizer. The detailed analysis of both docked molecules was conducted on the basis of interacting residues. 11-Hydroxytephrosin interacts with Glu90, Lys111, and Glu166 via hydrogen bonding, and several other residues participate in various other interactions (Figure 2A). At the same time, Torosaflavone A interacts with Lys111, Glu130, Glu166, Asp205, and Glu209 via hydrogen bonding, and several other residues participate in various other hydrophobic interactions (Figure 2B). Both compounds have several common interactions with PDPK1’s ATP-binding pocket residues, critical for its functional activity (Figure 2). Both compounds also share some common interactions with LY333531 (Figure 2C) [11]. Because 11-Hydroxytephrosin and Torosaflavone A bind to the ATP-binding pocket, their stability may prevent PDPK1 from accessing ATP, causing functional inhibition.

### 3.5. MD Simulations

MD simulations on docked complexes can competently improve docking models by accounting for the flexibility of protein and protein–ligand complexes [37,38]. Where experimental efforts fail, MD simulations can assist by filling in the structural specifics and conformational behavior of proteins [39]. All-atom MD simulations on PDPK1 and its complexes with 11-Hydroxytephrosin and Torosaflavone A were run in a solvent environment to analyze the conformational dynamics and stability of the docked complexes over time. PDPK1 docking complexes with the finest 11-Hydroxytephrosin and Torosaflavone A poses were created and employed in a 100 ns MD simulation. To obtain insights into the dynamics of PDPK1 before and after 11-Hydroxytephrosin and Torosaflavone A binding, the time evolution of various parameters was evaluated.

#### 3.5.1. Structural Changes and Compactness

**Figure 3:**
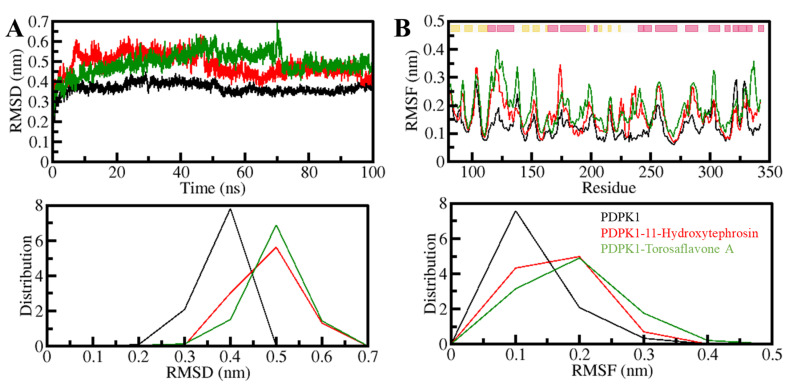
The structural dynamics of PDPK1 upon 11-Hydroxytephrosin and Torosaflavone A binding. (**A**) The RMSD plot of PDPK1 in complex with 11-Hydroxytephrosin and Torosaflavone A. (**B**) The RMSF plot of PDPK1 and its complex with 11-Hydroxytephrosin and Torosaflavone A. The top band at the RMSF plot shows the secondary structure assignments in PDPK1 where the sheet and helix are represented by yellow and pink, respectively. The lower panels show the value distribution as PDF.

To investigate protein structural variation over time, the root-mean-square deviations (RMSD) study is a valuable method [40]. The PDPK1 backbone was determined using the RMSD and RMSF analyses. The RMSD data of the PDPK1-11-Hydroxytephrosin and PDPK1-Torosaflavone A complexes were used to create time evolution charts of backbone aberrations during the simulation. Figure 3A shows the results of the simulation trajectory, which were then used to analyze the complex stability. However, certain random variations in the RMSD of ~0.2 nm could be detected in the plot, mainly spread up to 70 ns. Constant and modest oscillations indicate the system’s stability in the backbone atoms. Compared to the free state of PDPK1, the fluctuations were minimized in the ligand-bound states, as seen by the RMSF plot. During the 100 ns simulation track, all three systems appeared to have attained equilibrium and remained stable (Figure 3B). The RMSD and RMSF of the PDPK1-Torosaflavone A complex was more stable and equilibrated throughout the simulation than the PDPK1-11-Hydroxytephrosin complex. In the ligand-bound states of PDPK1, we also displayed the RMSD and RMSF distribution as a probability density function (PDF), demonstrating slightly higher values in the PDPK1 dynamics with decreased probability (Figure 3, lower panel). Overall, the RMSD and RMSF studies indicate that ligand-bound systems are stable and do not go through any large structural deviation during the simulation.

**Figure 4:**
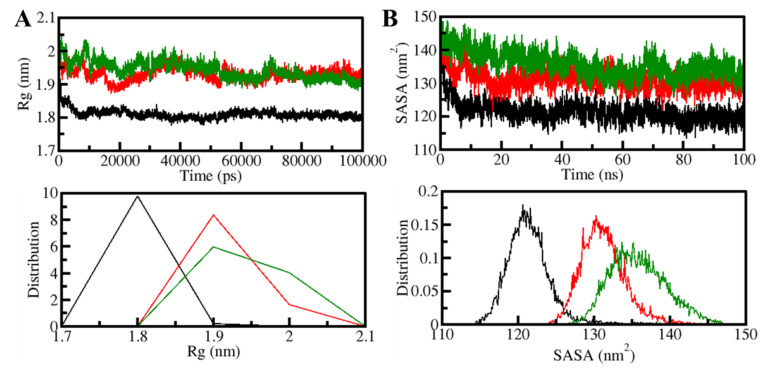
The structural compactness of PDPK1 upon 11-Hydroxytephrosin and Torosaflavone A binding. (**A**) The *R_g_* and (**B**) SASA plot of PDPK1 with 11-Hydroxytephrosin and Torosaflavone A binding.

The radius of gyration (*R_g_*) is the root-mean-square distance between the collective center of mass of a group of atoms and is related to the 3D-structure of a protein. *R_g_* is a commonly used measure to determine how compact a protein structure is [41]. The temporal evaluation of *R_g_* was used to determine the PDPK1 compactness in the free and ligand-bound forms. The compactness of three systems (PDPK1-apo, PDPK1-11-Hydroxytephrosin, and PDPK1-Torosaflavone A) was evaluated throughout the simulation. PDPK1, in the presence of 11-Hydroxytephrosin and Torosaflavone A, appeared to be steady between values of 1.8 and 2.0 nm throughout, according to the *R_g_* plot (Figure 4A). After 11-Hydroxytephrosin and Torosaflavone A binding, the folding dynamics of PDPK1 were slightly increased but continuously stable, according to the results. The PDF plot also revealed that the *R_g_* values for PDPK1 were slight after 11-Hydroxytephrosin and Torosaflavone A binding (Figure 4A, lower panel).

The surface area of a molecule accessible to its neighboring solvent is known as the solvent-accessible surface area (SASA) [42]. It has long been regarded as a crucial part of protein folding investigation [38]. The number of native connections and SASA analyses are useful in determining the degree of protein folding [42]. We investigated the time evolution of PDPK1’s SASA before and after 11-Hydroxytephrosin and Torosaflavone A binding. The graphic indicated a slight increment throughout the simulation but no significant changes in the SASA values. According to the SASA analysis, the PDPK1 structure looked stable during the simulation, even when 11-Hydroxytephrosin and Torosaflavone A were present (Figure 4B). The PDF likewise displayed a similar pattern with increased values in the SASA values of PDPK1 and its complexes with 11-Hydroxytephrosin and Torosaflavone A (Figure 4B, lower panel).

#### 3.5.2. Dynamics of Hydrogen Bonds

**Figure 5:**
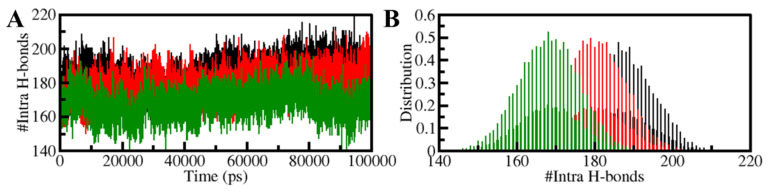
**Intramolecular hydrogen bonding.** (**A**) Hydrogen bonds formed intra-PDPK1. (**B**) The probability density function (PDF) of the intramolecular H-bonds within PDPK1.

The structural conformation of a protein is stabilized by intramolecular hydrogen bonding (H-bonds) and hydrophobic interactions [43]. H-bonds serve an essential function in the overall conformation of a protein [44]. Intramolecular H-bonds have long been used to investigate the conformational variations and compactness of the protein structure [45]. Over time, the number of H-bonds generated intramolecularly in PDPK1 was calculated using MD trajectories. The results allowed us to investigate the stability of intramolecular bonding in PDPK1 before and after 11-Hydroxytephrosin and Torosaflavone A binding. The number of H-bonds created within PDPK1 fluctuated before and after 11-Hydroxytephrosin and Torosaflavone A binding, as shown in Figure 5. The graph shows that the H-bonds that formed inside PDPK1 were long-lasting and contributed to the stability of the protein structure. A slight decrease in the intramolecular H-bonds inside the PDPK1-11-Hydroxytephrosin and PDPK1-Torosaflavone A complexes suggests lower compactness than the free PDPK1 structure, which comes from the use of some of the intramolecular space by the ligands, as seen in the PDF plot (Figure 5B).

**Figure 6:**
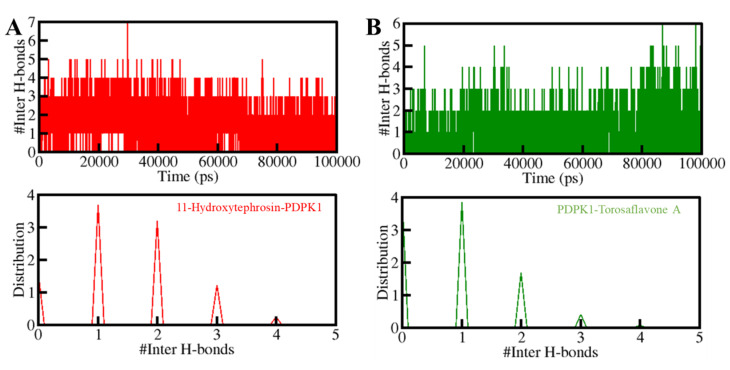
The H-bonds between PDPK1 and (**A**) 11-Hydroxytephrosin and (**B**) Torosaflavone A.

Furthermore, the time-evolution of the intermolecular H-bonds was also investigated to assess the consistency of the docked complexes of PDPK1 with 11-Hydroxytephrosin and Torosaflavone A. The average number of hydrogen bonds generated in the 11-Hydroxytephrosin-PDPK1 and Torosaflavone A-PDPK1 complexes was calculated to be three in each (Figure 6, upper panel). Due to the increased score of PDF, at least one hydrogen bond was constantly formed between both complexes (Figure 6, lower panel). A stable docked complex is regulated by the formation of intermolecular H-bonding between the protein and ligands. The results indicate that 11-Hydroxytephrosin and Torosaflavone A will not migrate from their original docking position on PDPK1.

#### 3.5.3. Principal Component Analysis and Free Energy Landscapes

**Figure 7:**
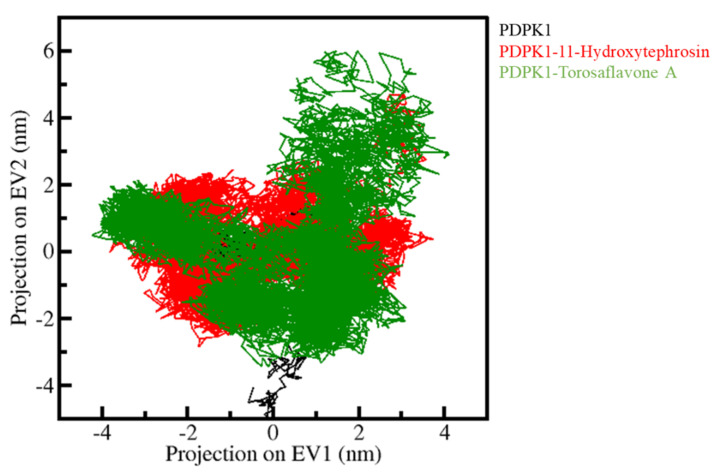
**PCA.** The 2D-projection of PDPK1, PDPK1-11-Hydroxytephrosin, and PDPK1-Torosaflavone A.

PCA was utilized to investigate the collective movements of PDPK1 and its docked complexes. We used the simulated trajectories for the PCA to investigate the conformational sampling of the PDPK1, PDPK1-11-Hydroxytephrosin, and PDPK1-Torosaflavone A complexes. Figure 7 depicts the conformational sampling in the essential subspace projected through the C_α_ atoms of all three systems (PDPK1, PDPK1-11-Hydroxytephrosin, and PDPK1-Torosaflavone A). The PDPK1-11-Hydroxytephrosin and PDPK1-Torosaflavone A complexes occupied the same essential subspace as PDPK1 in the free state, as shown in the graph. In both EVs, the PDPK1-11-Hydroxytephrosin complex was depicted in a subspace that was nearly identical to the free-PDPK1. The PDPK1-11-Hydroxytephrosin complex has less flexibility than the PDPK1-Torosaflavone A complex on both EVs, supporting the compactness study (Figure 6).

**Figure 8:**
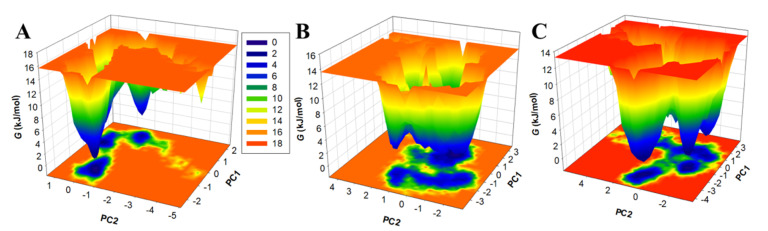
The FEL plots of the (**A**) free PDPK1, (**B**) PDPK1-11-Hydroxytephrosin, and (**C**) PDPK1-Torosaflavone A.

Nowadays, free energy landscapes (FELs) are used to describe the protein folding mechanism [35,46]. It has been exploited to investigate the stability of protein and protein–ligand complexes in solvents using MD simulation trajectories [35]. The energy minima and conformational landscape of the PDPK1, PDPK1-11-Hydroxytephrosin, and PDPK1-Torosaflavone A complexes were retrieved using two PCs. Figure 8 shows the contoured maps for the FELs of the PDPK1, PDPK1-11-Hydroxytephrosin, and PDPK1-Torosaflavone A complexes. The size and position of the phases restricted inside 2–3 stable global minima were slightly affected by 11-Hydroxytephrosin and Torosaflavone A binding with PDPK1, according to the FEL plots (Figure 8). In the FELs, the darker blue indicates a conformation with lower energy near the native states. PDPK1 appeared to be restricted within a massive single global minimum that expanded to 2–3 basins; according to the plot, PDPK1-11-Hydroxytephrosin and PDPK1-Torosaflavone A both developed nearly identical states, with a lower global minimum and 3–4 local basins with varied populations (Figure 8B,C). Overall, the MD simulation and essential dynamics of PDPK1 in complex with 11-Hydroxytephrosin and Torosaflavone A revealed their stability during 100 ns simulations with minimal conformational flipping.

## 4. Conclusions

Overall, our findings suggest that while PDPK1 is regarded as an essential therapeutic target due to its role as a positive regulator of cancer development, proliferation, and migration, natural compounds targeting PDPK1 can be employed to control pathways with anti-cancer effects. Understanding the complexities of the metabolic switching of cancer cells will aid in developing novel ways for successful and targeted therapy. Our method will be valuable in developing cancer therapies that leverage natural leads as powerful PDPK1 inhibitors as well as therapeutic models for metabolic reprogramming. We discovered two natural compounds, 11-Hydroxytephrosin and Torosaflavone A, that were effective PDPK1 modulators. The results showed that 11-Hydroxytephrosin and Torosaflavone A have higher affinities than LY333531 toward PDPK1. Both compounds shared common interactions with LY333531 and showed better ADMET and drug-like properties than LY333531. To determine the interaction between the elucidated compounds and PDPK1, molecular docking was used, revealing that the binding mechanism of both compounds was almost similar with several common interactions and was stable in the MD simulation studies. Our findings show that 11-Hydroxytephrosin and Torosaflavone A can act as promising inhibitory molecules of PDPK1 and can be used as a starting point for developing effective and selective PDPK1 inhibitors to slow cancer growth.

## Figures and Tables

**Table 1:** The selected top 50 molecules based on the binding affinity toward PDPK1.

S. No.	Compound ID	Affinity (kcal/mol)
1.	CID_24901683	−11.6
2.	CID_3822	−10.8
3.	CID_442851	−10.5
4.	CID_101277405	−10.4
5.	CID_44257658	−10.3
6.	CID_13846202	−10.2
7.	CID_4970	−10.2
8.	CID_101746	−10.2
9.	CID_5245667	−10.1
10.	CID_102060338	−9.9
11.	CID_44257628	−9.9
12.	CID_73393	−9.8
13.	CID_480799	−9.8
14.	CID_102267534	−9.8
15.	CID_120734	−9.7
16.	CID_440583	−9.7
17.	CID_196979	−9.7
18.	CID_4437370	−9.7
19.	CID_10925304	−9.7
20.	CID_5318619	−9.7
21.	CID_14033985	−9.7
22.	CASID_53777-78-9	−9.6
23.	CID_6770	−9.6
24.	CASID_22296-77-1	−9.6
25.	CID_124069	−9.6
26.	CID_147329	−9.6
27.	CID_124050	−9.6
28.	CID_443716	−9.6
29.	CID_197775	−9.6
30.	CID_601058	−9.6
31.	CID_641765	−9.6
32.	CID_5281353	−9.6
33.	CID_5316096	−9.6
34.	CID_5281406	−9.6
35.	CID_101277371	−9.6
36.	CID_101667973	−9.6
37.	CASID_13241-28-6	−9.5
38.	CID_120698	−9.5
39.	CID_114909	−9.5
40.	CID_5459059	−9.5
41.	CID_6510278	−9.5
42.	CID_5281867	−9.5
43.	CASID_74148-50-8	−9.4
44.	CID_101595	−9.4
45.	CID_146680	−9.4
46.	CID_10336244	−9.4
47.	CID_5281809	−9.3
48.	CASID_94418-50-5	−9.3
49.	CASID_64191-02-2	−9.2
50.	CID_5154	−9.2
51.	LY333531	−8.3

**Table 2:** The ADMET parameters of the selected molecules using the pkCSM Web tool.

Compound ID	Compound Name	GI Absorption	Water Solubility (log mol/L)	BBB Permeability (log BB)	CYP2D6 Inhibitor	OCT2 Substrate	AMES
CID_5318619	Isoononin	High	−3.39	−1.27	No	No	No
CID_44257658	Torosaflavone A	High	−2.90	−1.37	No	No	No
CID_13846202	11-Hydroxytephrosin	High	−3.89	−0.18	No	No	No
LY333531	Ruboxistaurin	High	−4.81	−0.48	No	No	Yes

**Table 3:** The top 10 relevant biological properties of the elucidated compounds.

Compound	Pa	Pi	Biological Activity
11-Hydroxytephrosin	0.934	0.004	Antineoplastic
	0.904	0.003	Antineoplastic (non-small cell lung cancer)
	0.857	0.003	Prostate cancer treatment
	0.822	0.003	Antineoplastic (ovarian cancer)
	0.799	0.004	Chemopreventive
	0.775	0.014	TP53 expression enhancer
	0.654	0.020	Apoptosis agonist
	0.644	0.016	Kinase inhibitor
	0.542	0.005	Antioxidant
	0.540	0.015	Antineoplastic (breast cancer)
Torosaflavone A	0.929	0.005	Membrane integrity agonist
	0.889	0.006	TP53 expression enhancer
	0.860	0.006	Antineoplastic
	0.840	0.003	Chemopreventive
	0.832	0.003	Cardioprotectant
	0.827	0.005	Kinase inhibitor
	0.821	0.005	Anticarcinogenic
	0.800	0.004	Hepatoprotectant
	0.730	0.012	Apoptosis agonist
	0.725	0.009	Antifungal
LY333531 (ruboxistaurin)	0.869	0.007	Antineurotic
	0.823	0.009	Antineoplastic
	0.788	0.004	Chemo preventive
	0.745	0.011	Apoptosis agonist
	0.720	0.044	Hepatoprotectant
	0.710	0.012	TP53 expression enhancer
	0.707	0.010	Kinase inhibitor
	0.699	0.008	Anticarcinogenic
	0.638	0.004	Antidiabetic
	0.612	0.010	Antioxidant

## Data Availability

Not applicable.
